# MG132 Attenuates the Replication of Classical Swine Fever Virus *in vitro*

**DOI:** 10.3389/fmicb.2020.00852

**Published:** 2020-06-03

**Authors:** Yuming Chen, Shuangqi Fan, Mengpo Zhao, Keke Wu, Erpeng Zhu, Shengming Ma, Wencheng He, Shaofeng Deng, Hailuan Xu, Jingyuan Zhang, Hongxing Ding, Lin Yi, Mingqiu Zhao, Jinding Chen

**Affiliations:** ^1^College of Veterinary Medicine, South China Agricultural University, Guangzhou, China; ^2^Guangdong Laboratory for Lingnan Modern Agriculture, Guangzhou, China

**Keywords:** classical swine fever virus, MG132, 26S proteasome, JAK-STAT pathway, STAT1

## Abstract

The 26S proteasome, in charge of intracellular protein degradation, plays significant roles in the modulation of various cellular activities as well as in the interplay between virus and host. However, studies about the relationship between 26S proteasome and classical swine fever virus (CSFV) is limited up to now. MG132 is a proteasome inhibitor and has been extensively used in studies about replication of many viruses. Herein, we investigated the role of MG132 in CSFV replication and results showed that MG132 significantly decreased virus titers and viral RNA copies in CSFV-infected PK-15 cells. Further studies demonstrated that MG132 upregulated the expression of several interferon-stimulated genes (ISGs), in CSFV-infected cells. Since the activation of ISGs is controlled by the JAK-STAT signal pathway, we next examined the effect of MG132 on the expression and localization of key molecular STAT1 in the infected cells using Western blot and confocal laser scanning microscopy, respectively. Results showed that CSFV infection and viral NS4A protein decreased the protein level of STAT1, and MG132 promoted the accumulation of STAT1 in the nucleus of cells adjacent to the CSFV-infected cells. Besides, MG132 did not affect the expressions of *IFN*-α, *STAT1*, *Mx1*, *OAS1*, and *PKR* genes in cells without CSFV. In conclusion, we identify that MG132 significantly inhibits CSFV replication *in vitro*, in which the activation of the JAK-STAT pathway and the subsequent upregulation of expressions of ISGs might play significant roles, providing a potential preventive method for CSF.

## Introduction

The 26S proteasome, which consists of a 20S catalytic core and two 19S regulatory subunits at the end (Kim et al., 2011), plays important roles in the regulation of various fundamental cellular processes including cell cycle, cell apoptosis, signal transduction, innate immune, etc. (Goldberg, 2007). There is a close relationship between 26S proteasome and the ubiquitin reaction cascade, since most proteins degraded by 26S proteasome are modified by ubiquitin. 26S proteasome together with the ubiquitin reaction cascade constitutes the ubiquitin proteasome system (UPS), mediating the degradation of both cellular and viral proteins. The 26S proteasome has been reported to be involved in the infection of various viruses by regulating levels of viral proteins and relevant cellular proteins (Rajsbaum and Garcia-Sastre, 2013; Davis and Gack, 2015; Luo, 2016).

Classical swine fever (CSF), caused by classical swine fever virus (CSFV), is a porcine infectious disease characterized by highly contagious and often fatal outcome (Vilcek and Nettleton, 2006; Luo et al., 2014). CSFV is an enveloped single-stranded RNA virus that belongs to the genus *Pestivirus* within the family Flaviviridae (Ruggli et al., 1996). The genome of CSFV encodes a viral polyprotein which could be cleaved to form four structural proteins (E^rns^, E1, E2, and C) and eight non-structural proteins (N^pro^, p7, NS2, NS3, NS4A, NS4B, NS5A, and NS5B) by enzymes (Lamp et al., 2011; Ji et al., 2015).

Usually, innate immune response is activated due to virus infection, followed by the release of a variety of antiviral and inflammation-inducing molecules including interferons (IFNs), proinflammatory cytokines, and chemokines (Borden et al., 2007; Nie and Wang, 2013). Upon secretion, IFN binds to the receptors on cell surface, activates JAK1 and Tyk2, and leads to phosphorylation of STAT1 and STAT2 (Stark and Darnell, 2012). pSTAT1 either dimerizes itself or with pSTAT2, forms a complex with IFN α/β-stimulated gene factor 3 (ISGF3), and subsequently moves to the nucleus (Villarino et al., 2017). The complex binds to the IFN-stimulated response elements, inducing transcription of more than 100 IFN-stimulated genes (ISGs; Sen and Peters, 2007; Sadler and Williams, 2008). Most of the ISGs-encoded proteins could play strong antiviral roles by up-regulating the cellular antiviral condition in many ways (Sadler and Williams, 2008). Among them, Mx1, GBP1, and OASL proteins have been identified to strongly inhibit CSFV replication (Li et al., 2016; Li L. F. et al., 2017; Zhou et al., 2018). Meanwhile, CSFV has developed various ways to attenuate the host innate immune system, which contributes to consistent viral replication (Ruggli et al., 2003, 2005; Xia et al., 2007; Doceul et al., 2008; Chen et al., 2012).

The 26S proteasome plays multiple roles in the modulation of viral replication. As a cellular machine of protein degradation, 26S proteasome could modulate virus replication via degradation of viral proteins (Luo, 2016). As to CSFV, viral proteins N^pro^, C, and p7 have been identified to be degraded by the 26S proteasome and affect CSFV replication, but the roles of degradations of the viral proteins in virus replication remains unknown (Seago et al., 2010; Gladue et al., 2012; Lin et al., 2014; Chen et al., 2019). Meanwhile, viruses have developed methods to take use of 26S proteasome for its persistent replication (Luo, 2016). A growing number of viruses are found to weaponize the ubiquitin modification system to degrade cellular proteins, which serve as restriction factors during virus replication, contributing to their consistent replication (Luo, 2016). Besides, the IFN signal pathway and IFN-induced JAK-STAT pathway are widely modulated by the 26S proteasome via regulating the levels of critical molecules (Davis and Gack, 2015; Heaton et al., 2016; Nan et al., 2017). Studies about the relation of CSFV and 26S proteasome are limited up to now and it will be of great significance to reveal the impact of 26S proteasome on CSFV replication.

Up to now, several types of proteasome inhibitors have been discovered or synthesized (Kisselev et al., 2012). MG132, a potent covalent inhibitor of the aldehyde proteasome pathway, forms a hemiacetal with the hydroxyl of the active site threonines and thus inhibits proteasome function (Kisselev et al., 2012). MG132 is widely used in studies about viral infection and replication. MG132 has been identified to play inhibitory roles in replication of herpes simplex virus type 1 (HSV-1; Delboy et al., 2008), human cytomegalovirus (HCMV; Kaspari et al., 2008), human coxsackievirus B3 (CVB3; Si et al., 2008), hepatitis C virus (HCV), severe acute respiratory syndrome coronavirus (SARS-CoV; Schneider et al., 2012), porcine circovirus type 2 (PCV2; Cheng et al., 2014), bovine herpesvirus 1 (BoHV-1; Fiorito et al., 2017), etc.

Considering the significance of 26S proteasome in the modulation of various cellular activities and the replication of many viruses, we try to investigate the impact of MG132 on CSFV and to explain the underlying mechanism.

## Materials and Methods

### Antibodies

Mouse anti-E2 antibody (9011, Median), rabbit anti-STAT1 antibody (AF0288, Beyotime), rabbit anti-pSTAT1 antibody (7649, Cell Signaling Technology), mouse anti-EGFP antibody (AG281, Sigma), mouse anti-tubulin antibody (AT819, Beyotime), HRP-conjugated goat anti-mouse IgG antibody (A0216, Beyotime), goat anti-mouse IgG (whole molecule)–fluorescein isothiocyanate (FITC) antibody (F0257, Sigma), and goat anti-rabbit IgG (whole molecule)–tetramethyl rhodamine isocyanate (TRITC) antibody (T6778, Sigma) were used in this study.

### Cells and Virus Infection

PK-15 cells (a porcine kidney cell line; ATCC, CCL-33) were grown in Dulbecco’s modified Eagle’s medium (DMEM) supplemented with 10% fetal bovine serum (FBS). Cells were cultured at 37°C in a 5% CO_2_ incubator. Cells were incubated with CSFV strain Shimen at varying multiplicities of infection (MOIs) for 2 h at 37°C. The viral inoculum was removed later and cells were maintained with DMEM containing 2% FBS. At 24 and 48 hours post infection (hpi), the supernatants were collected to detect viral infectivity titers. The cell monolayers were lysed to detect the relative mRNA level of viral genomic copies by RT-PCR.

### Plasmids Construction and Transfection

The *NS4A* gene of CSFV strain Shimen (GenBank accession no. AF092448.2) was cloned into the pEGFP-C1 vector (Clontech) with the *Xho*I and *BamH*I restriction enzymes to generate the plasmid pEGFP-NS4A encoding EGFP-NS4A fusion protein. Plasmid encoding NS4AΔ4, a truncated form of NS4A protein, was generated from pEGFP-NS4A by conventional PCR with the primers listed in Table 1. All plasmids were verified by sequencing.

**TABLE 1:** Primers used in this study.

Primer name	Sequence (5′-3′)	Use
Q-CSFV-F	CCTGAGGACCAAACACATGT	Amplification of CSFV NS5B
Q-CSFV-R	TGGTGGAAGTTGGTTGTGTCTG	
Q-Mx1-F	GAACGAAGAAGACGAATGGAAGG	Amplification of Mx1
Q-Mx1-R	GATGCCAGGAAGGTCTATGAGG	
Q-PKR-F	GGGGAAGATGGGCCTGAAAA	Amplification of PKR
Q-PKR-R	GGATGGTGGGTCAGCATTCA	
Q-OAS1-F	ACCTGAAGTACGTGAAAGCCA	Amplification of OAS1
Q-OAS1-R	ACGAGGCCTCTGTCCAAATG	
Q-STAT-F	CAGCTGAACATGCTGGGAGA	Amplification of STAT1
Q-STAT-R	GCTGCTGGTCCTTTAGCAGA	
Q-GAPDH-F	TGGAGTCCACTGGTGTCTTCAC	Amplification of GAPDH
Q-GAPDH-R	TTCACGCCCATCACAAACA	
Q-IFNA-F	CTCAGCCAGGACAGAAGCA	Amplification of IFN-α
Q-IFNA-R	TCACAGCCCAGAGAGCAGA	
NS4A-F	CAGCTCGAGCTATGTCAACAGCTGA	Amplification of NS4A
NS4AΔ4-F	ACTCGAGCTATGAGGCATATACCAG	Amplification of NS4AΔ4
NS4A-R	CGGGATCCTCATAGCTCCTTCAATTC	Amplification of NS4A and NS4AΔ4

Cells in 12-well plates were transfected with the indicated plasmids (1.5 μg per well) using Lipo6000^TM^ transfection reagent (C0528; Beyotime) according to the manufacturer’s instructions. At 4 hours post transfection (hpt), the transfection mixture was replaced with DMEM supplemented with 2% FBS. Cells were lysed at the indicated time points followed by Western blot analysis.

### Biochemical Intervention and Cell Viability

MG132 (A2585, Apexbio) is stored at -20°C at a concentration of 10 mM diluted in dimethyl sulfoxide (DMSO). MG132 and DMSO were diluted with DMEM containing 2% FBS when using. PK-15 cells were treated with MG132 after the incubation of CSFV until the end of the experiment. The same volume of DMSO was used as a control.

To detect the effects of MG132 or DMSO on cells, a cell viability assay was carried out using cell counting kit 8 (CCK-8; catalog no. CK04; Beyotime) according to the manufacturer’s instructions.

### Quantitative Real-Time Polymerase Chain Reaction

Total RNA was extracted from cells and was reverse transcribed into the cDNA using Moloney murine leukemia virus reverse transcriptase (2641A, TaKaRa) according to the manufacturer’s instructions. Gene expression was quantified by quantitative real-time polymerase chain reaction (qRT-PCR) with TB Green Premix Ex Taq II (RR820A, TaKaRa) in the CFX96 real-time PCR system (Bio-Rad). Primers used to detect CSFV NS5B, GAPDH, STAT1, Mx1, OAS1, PKR, and IFN-α are listed in Table 1. Primers for detection of NS5B, GAPDH, IFN-α and Mx1 are synthesized according to previous studies (Pei et al., 2016; Li L. F. et al., 2017). The relative abundance of each target was obtained by normalization with endogenous GAPDH.

### Virus Titers

PK-15 cells were cultivated in a 96-well plate and were inoculated with 10-fold serial dilutions of virus. Cells were incubated at 37°C for 2 days. Cell medium was discarded and cells were fixed with absolute ethanol at room temperature for 20 min. Viruses were detected by an immunofluorescence assay (IFA) using mouse anti-CSFV E2 antibody (1:200) and goat anti-mouse IgG (whole molecule)–FITC antibody (1:200). Virus titers were calculated by the Reed–Muench method (Reed and Muench, 1938) and are expressed as median tissue culture infective doses (TCID_50_) per 0.1 ml.

### Confocal Microscopy Assays

Confocal microscopy was performed at the indicated time points. Briefly, cells were fixed with absolute ethanol for 20 min and then rinsed with PBS. After rinsing, cells were incubated with the anti-E2 or anti-STAT1 primary antibodies (1:100) at 37°C for 1 h. After rinsing with PBS, cells were then incubated with goat anti-mouse IgG (whole molecule)–FITC antibody (1:100) and goat anti-rabbit IgG (whole molecule)–tetramethyl rhodamine isocyanate (TRITC) antibody (1:100) at 37°C for 1 h. Cells were rinsed with PBS and stained with 4,6-diamidino-2-phenylindole (DAPI) at room temperature (RT) for 10 min. Cells were examined using a Leica SP2 confocal system (Leica Microsystems, Germany).

### Western Blot Analysis

Cell supernatant was discarded and cells were washed three times with PBS. Moderate amount of cell lysis buffer containing proteasome inhibitors and phosphatase inhibitors (Beyotime, P1045) was added to the cell monolayer and incubated for 15 min on ice. Cell lysates were clarified by centrifugation at 12,000 *g* for 5 min. The precipitated protein samples were mixed with 5× protein loading buffer, boiled at 100°C for 10 min and subjected to SDS-PAGE. Proteins were separated and transferred to a PVDF membrane (Roche). The nonspecific antibody binding sites were blocked with 4% bovine serum albumin (BSA) diluted in PBS. Membranes were incubated with anti-tubulin (1:2000), anti-STAT1 (1:1000), anti-pSTAT1 (1:1000), or anti-EGFP (1:2000) primary antibodies diluted in PBS at 4°C overnight, and HRP-conjugated goat anti-mouse IgG secondary antibody (1:1000) diluted in PBS at 37°C for 1 h. Protein bands were detected with the ECL Plus kit (Beyotime, P0018) by luminescent image (Tanon 6600).

### Statistical Analysis

Statistical analyses were performed using GraphPad Prism software. Student’s *t* test was used to compare data in this study. A *P* value of <0.05 was considered significant.

## Results

### Effect of MG132 on Cell Viability

**FIGURE 1:**
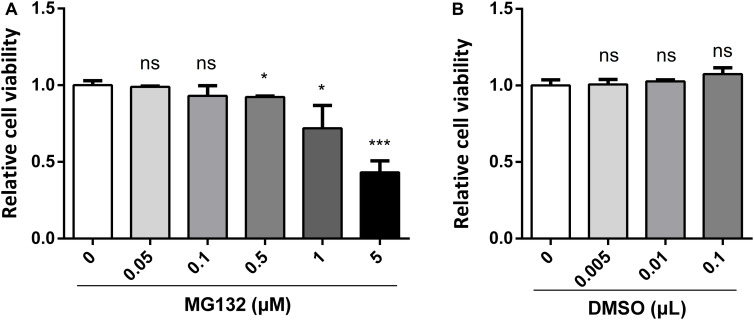
Effects of different concentrations of MG132 or DMSO on viability of PK-15 cells. Different concentrations of MG132 **(A)** or DMSO **(B)** were added to the cell culture medium and incubated for 48 h, respectively. Cell viability was detected by CCK-8 assay. Results were shown as relative cell viability.

MG132, one of the potent inhibitors of 26S proteasome, has been widely used in studies of infection and replication of viruses, while its role during CSFV infection is not known yet. Therefore, we decided to assess the role of MG132 in CSFV replication in PK-15 cells. Firstly, we tested the influence of different concentrations of MG132 on the viability of PK-15 cells using CCK-8 assay. Results showed that cell activity decreased with increasing concentrations of MG132 (Figure 1A). Since MG132 of 0.5 and 1 μM significantly decreased cell viability compared with mock-treated cells, a relatively high concentration of MG132 (0.1 μM) that has no obvious impact on cell viability was firstly selectively used in this study. The effects of different volumes of DMSO on cell viability were also analyzed and results showed that DMSO has no obvious effect on PK-15 cells compared with no treated cells (Figure 1B).

### MG132 Inhibits CSFV Replication in PK-15 Cells

**FIGURE 2:**
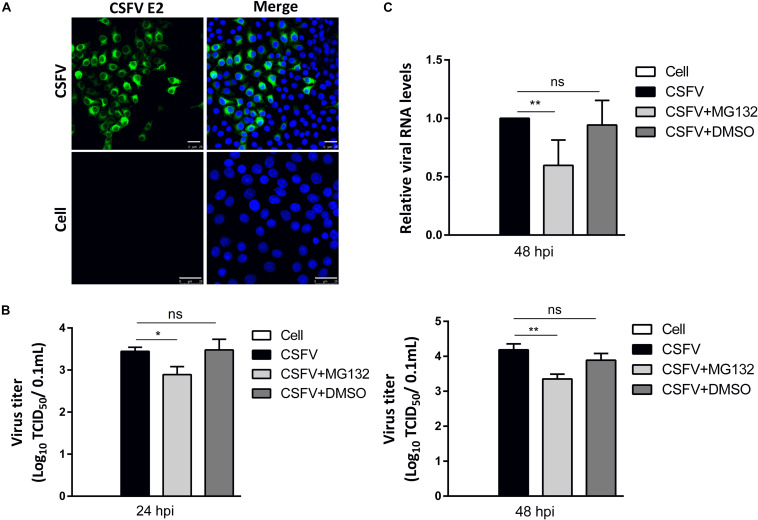
MG132 inhibits CSFV replication in PK-15 cells. **(A)** Detection of viral E2 protein in CSFV-infected cells by confocal microscopy. PK-15 cells were infected with CSFV at a MOI of 0.1 for 48 h. Confocal microscopy assay was performed to detect viral E2 protein (green) with an E2-specific antibody. Cellular nucleus was stained with DAPI and shown in blue. Uninfected PK-15 cells were used as controls. Scale bar, 25 μm. **(B,C)** Relative viral genomic copies and virus titers of CSFV. PK-15 cells were infected with CSFV of 0.1 MOI for 2 h at 37°C. After incubation, virus was discarded and cells were treated with 0.1 μM MG132, same volume of DMSO or left untreated. **(B)** The CSFV genomic RNA copy numbers at 48 hpi were determined by using qRT-PCR. Data in the bar plot are representative of six biological replicates. **(C)** The viral titers in the supernatant at 24 and 48 hpi were determined and presented as TCID_50_. Unless noted, data in all bar plots are shown as mean ± SD and representative of three biological replicates. *P < 0.05; **P < 0.01; ns, not significant.

PK-15 cells, a porcine kidney cell line, were infected with CSFV strain Shimen at a MOI of 0.1. Viral structural E2 protein was detected by confocal microscopy using mouse anti-E2 antibody and was shown in green (Figure 2A). Uninfected cells were used as controls. PK-15 cells in a 12-well plate were infected with 0.1 MOI of CSFV for 2 h, and further cultured in maintenance medium containing MG132 at a final concentration of 0.1 μM. The same volume of DMSO was used as a control. Classical swine fever virus titers in the supernatant at 24 and 48 hpi, as well as viral RNA levels at 48 hpi in cells, were detected using TCID_50_ and qRT-PCR, respectively. Compared with no treated cells, 0.1 μM MG132 decreased CSFV RNA level by 1.7-fold at 48 hpi, and virus titers by 3.5-fold at 24 hpi and by 6.9-fold at 48 hpi (Figures 2B,C), showing the strong inhibitory effect of MG132 on CSFV replication. In addition, DMSO had no obvious effect on both CSFV RNA copies and virus titers compared with no treated cells.

### MG132 Upregulates the Expressions of Several ISGs in CSFV Infected Cells

**FIGURE 3:**
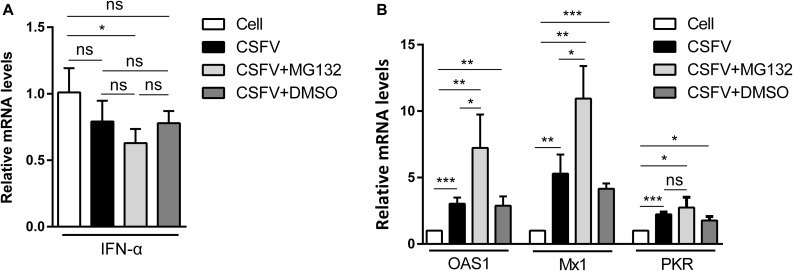
Relative mRNA levels of *IFN*-α, *Mx1*, *PKR*, and *OAS1*. **(A,B)** PK-15 cells were uninfected (Cell) or infected with CSFV followed by treated with MG132, DMSO, or left untreated as described above. The mRNA levels of *IFN*-α **(A)**, *Mx1*, *PKR*, and *OAS1* **(B)** at 48 hpi were determined by qRT-PCR. The relative abundance of each target was obtained by normalization with GAPDH. Data in the bar plot are representative of three biological replicates. *P < 0.05; **P < 0.01; ***P < 0.001; ns, not significant.

Since IFN-α, an important cytokine in the modulation of virus replication, and IFN-α-induced ISGs play great inhibitory roles in CSFV replication (Li et al., 2013, 2016), we further analyzed that whether MG132 could affect the levels of IFN-α and ISGs in CSFV-infected cells. PK-15 cells were uninfected (Cell) or infected with CSFV, and treated with MG132, DMSO, or left untreated as described above. The qRT-PCR assay was conducted to detect mRNA levels of *IFN*-α, *OAS1*, *Mx1*, and *PKR* genes. The mRNA expression of *IFN*-α was downregulated in MG132-treated cells in contrast with uninfected cells, and there is no difference between MG132, DMSO, and untreated groups (Figure 3A). We identified that CSFV infection promoted the transcription levels of *Mx1*, *PKR*, and *OAS1*, and MG132 further upregulated expressions of *Mx1* and *OAS1* compared with the CSFV group, though PKR was not significantly affected (Figure 3B). Since ISG*s*-encoded proteins play important inhibitory roles in CSFV replication (Li et al., 2016; Li L. F. et al., 2017; Zhou et al., 2018), we assume that the upregulation of ISGs may be a strategy utilized by MG132 to inhibit CSFV replication. Considering that *IFN*-α was downregulated in CSFV-infected cells in the presence of MG132, the upregulation of ISGs expression was probably not caused by IFN-α. While the transcription of ISGs is controlled by the JAK-STAT pathway which is influenced by many cytokines, the way that MG132 modulates ISGs needs further investigation.

### High Doses of MG132 Decrease CSFV Replication and Upregulate Expression Levels of Several ISGs

**FIGURE 4:**
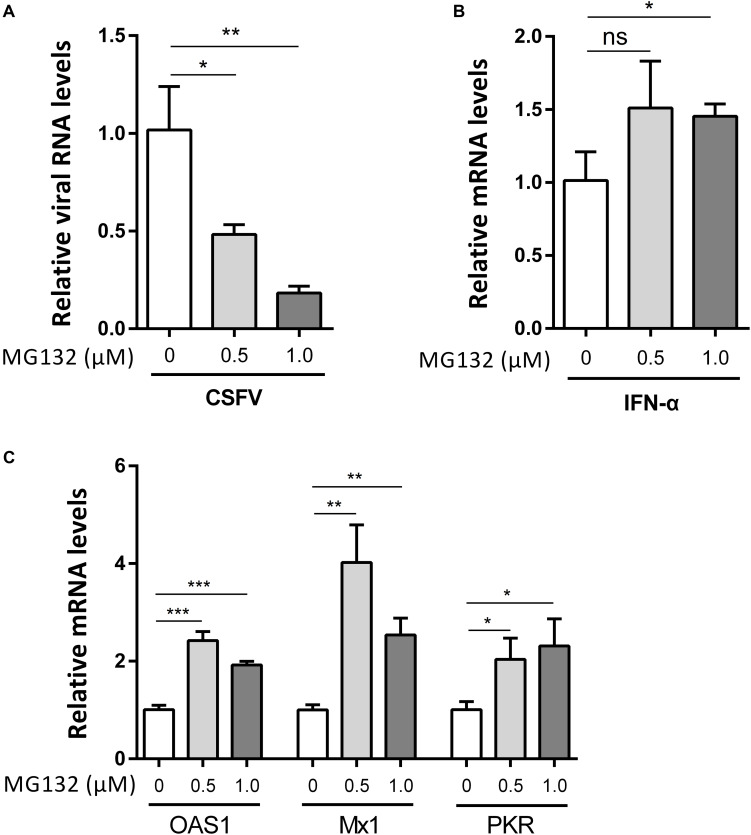
High doses of MG132 inhibit CSFV replication and upregulate the expression of *OAS1, Mx1*, and *PKR*. **(A–C)** PK-15 cells were infected with CSFV at a MOI of 1 followed by treatment of 0.5 μM MG132, 1.0 μM MG132, or DMSO, respectively. The CSFV genomic RNA copy numbers **(A)** at 24 hpi were determined by qRT-PCR. The mRNA levels of *IFN*-α **(B)** and several ISGs **(C)** at 24 hpi were determined by qRT-PCR. Data in the bar plot are representative of three biological replicates. *P < 0.05; **P < 0.01; ***P < 0.001; ns, not significant.

We used higher concentrations of MG132 of 0.5 and 1.0 μM to further confirm the effect of MG132 on CSFV replication and ISGs levels. Cells infected with CSFV followed by treatment of DMSO were used as controls. Results showed that 0.5 μM MG132 decreased CSFV RNA level by 2.1-fold, and 1.0 μM MG132 decreased CSFV RNA level by 5.7-fold (Figure 4A). The mRNA levels of *IFN*-α was increased by MG132 of 1.0 μM (Figure 4B). Besides, mRNA levels of *OAS1*, *Mx1*, and *PKR* were all significantly upregulated in cells treated with 0.5 or 1.0 μM MG132 compared with cells treated with DMSO (Figure 4C), showing that MG132 could stimulate the expression of ISGs in CSFV-infected cells.

### CSFV Infection and Viral Protein NS4A Decrease the Expression of STAT1

**FIGURE 5:**
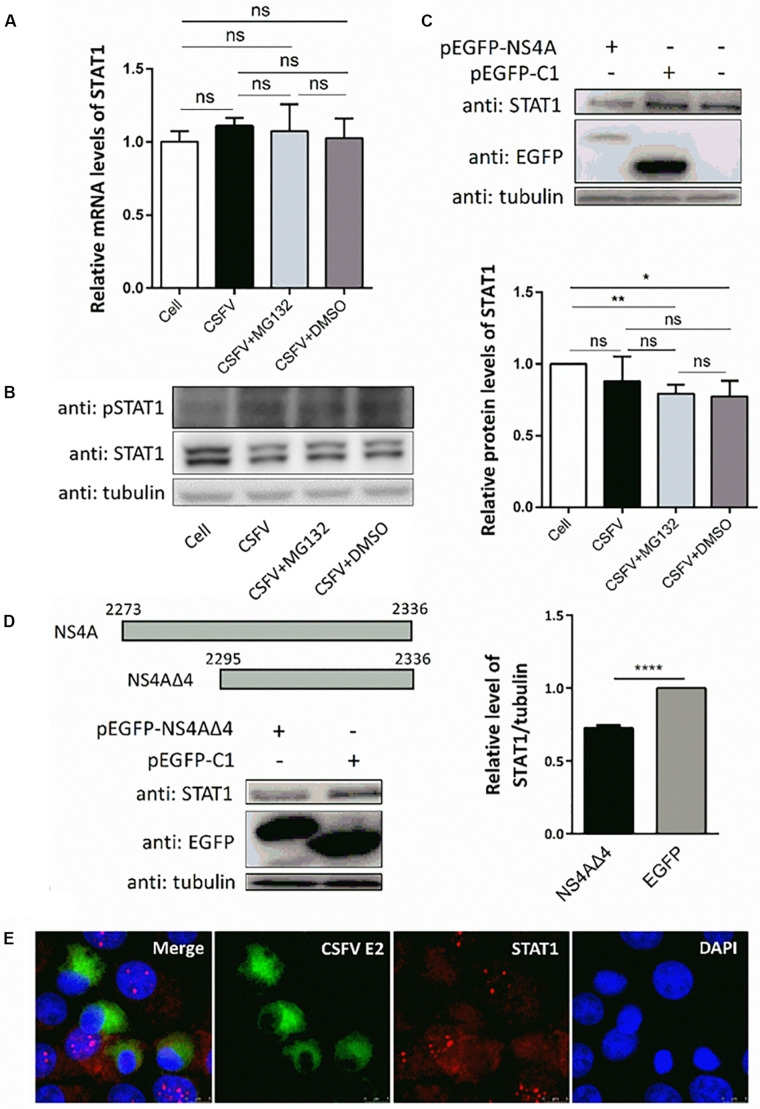
Effects of CSFV infection and viral NS4A expression on STAT1 and pSTAT1. **(A,B)** Effect of CSFV infection and MG132 treatment on expression of STAT1 and pSTAT1. PK-15 cells were uninfected (Cell) or infected with CSFV of 0.1 MOI, and treated with 0.1 μM MG132, DMSO, or left untreated. The mRNA levels **(A)** and protein levels **(B)** of STAT1 and pSTAT1 at 48 hpi were detected by qRT-PCR and Western blot, respectively. Relative protein level of STAT1 to tubulin was shown. **(C,D)** Effects of viral NS4A protein and its truncated NS4AΔ4 protein on STAT1 expression. PK-15 cells were transfected with plasmids pEGFP-NS4A, pEGFP-NS4ΔA, and pEGFP-C1 or left untreated. **(C)** Protein levels of EGFP-NS4A, EGFP, and STAT1 at 12 h post transfection (hpt) were detected by Western blot with the indicated antibodies. **(D)** Schematic representation of full-length NS4A and truncated NS4AΔ4 with the numbers above showing the starting and ending residues. Protein levels of EGFP-NS4ΔA, EGFP, and STAT1 at 24 hpt were detected by Western blot. Relative protein level of STAT1 to tubulin was shown. Tubulin was used as a loading control. Protein band intensity was evaluated with Image J software. **(E)** Effect of CSFV infection on STAT1 shown by confocal microscopy. Data in the bar plot are representative of three biological replicates. *P < 0.05; **P < 0.01; ***P < 0.001; ****P < 0.0001; ns, not significant.

Since transcription of ISGs is governed by the JAK-STAT pathway, we then tested the expression levels of key regulator STAT1 and pSTAT1 in PK-15 cells treated as described above. Compared with uninfected cells, CSFV infection had no obvious effect on the mRNA levels of *STAT1* (Figure 5A), but downregulated the protein level of STAT1 slightly (Figure 5B). Consistent with the upregulation of ISGs, expression of pSTAT1 was upregulated expectedly due to CSFV infection, while MG132 seemed to have no obvious effect on the expression of pSTAT1 (Figure 5B). To explore the underlying mechanism of the downregulation of STAT1 in CSFV-infected cells, plasmids encoding several viral proteins fused with an EGFP tag were constructed and transfected in PK-15 cells. EGFP-NS4A protein was found to suppress the expression of STAT1 in comparison with the EGFP and mock treated groups (Figure 5C). Furthermore, a truncated NS4A protein, NS4AΔ4, that could express at a relatively high level was also identified to downregulate the expression of STAT1 (Figure 5D). Results of the confocal microscopy assay showed that less signal (red) of STAT1 is observed in CSFV E2 positive cells (green) in a part of CSFV-infected cells compared with the E2 negative cells (Figure 5E).

### MG132 Induces the Accumulation of STAT1 in Surrounding Cells of CSFV-Infected Cells

**FIGURE 6:**
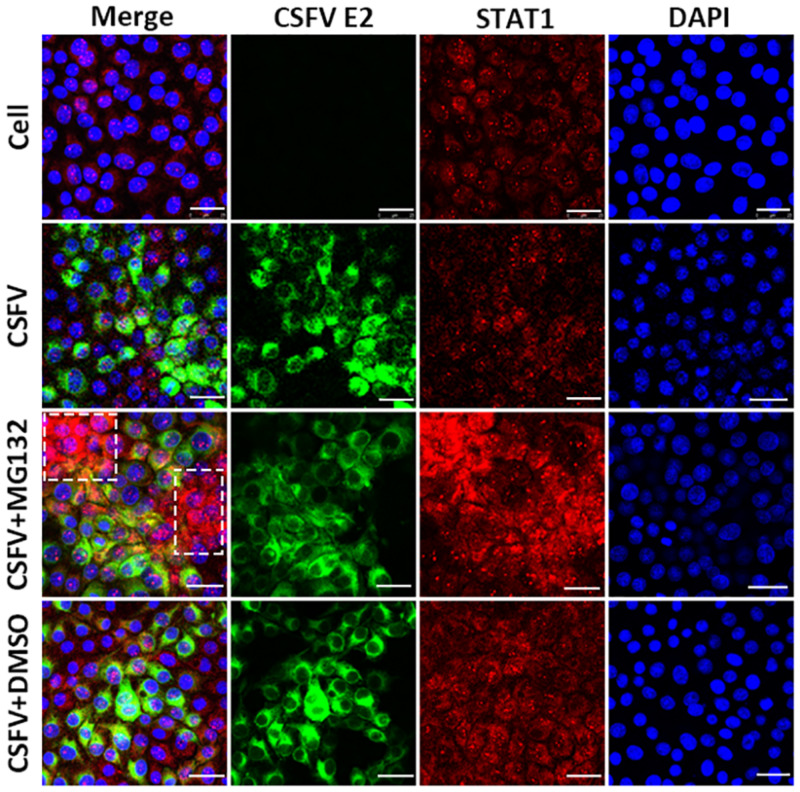
Effect of MG132 on the localization of STAT1 detected by confocal microscopy. PK-15 cells were uninfected (Cell) or infected with CSFV, and treated with 0.1 μM MG132 or DMSO or left untreated. At 48 hpi, STAT1 protein and viral E2 protein were detected with anti-STAT1 and anti-E2 primary antibodies, and the corresponding TRITC- and FITC-conjugated secondary antibodies, respectively. Cellular nucleus was stained with DAPI and shown in blue. The accumulation region of STAT1 in the MG132 treatment group was shown with a white dotted box. Scale bar, 25 μm.

Since the translocation of pSTAT1 from cytoplasm to nucleus is critical for the activation of the JAK-STAT pathway, the localization of STAT1 was then observed by laser scanning microscope in our study. Nuclear STAT1 protein was detectable in both mock- and CSFV-infected cells at 48 hpi, and CSFV seemed to have no obvious influence on the distribution of STAT1 protein (Figure 6). Notably, in contrast with DMSO and no treated cells, MG132 significantly induced the accumulation of STAT1 in the nucleus of certain uninfected nearby cells of CSFV-infected cells (shown with a dotted box), which may indicate a high activation level of the JAK-STAT pathway in these cells.

### MG132 Does Not Affect the Expressions of IFN-α, STAT1, and ISGs in Cells Without CSFV

**FIGURE 7:**
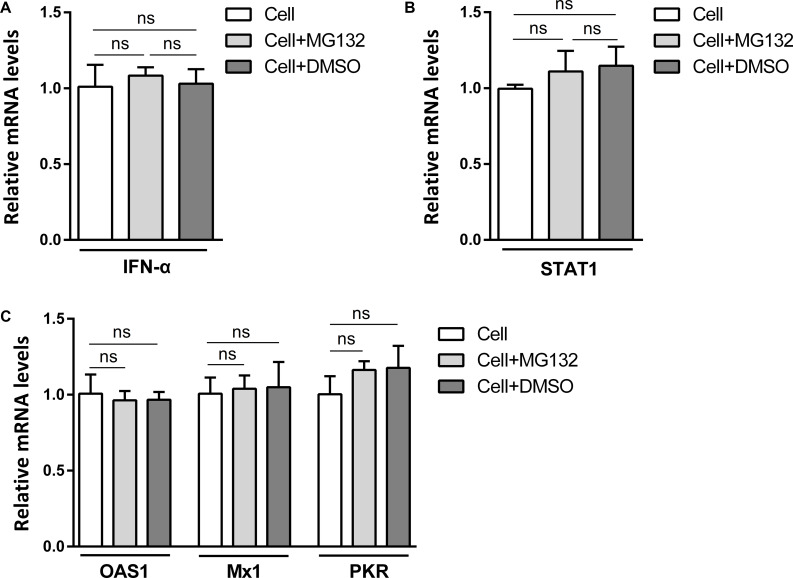
MG132 does not affect levels of *IFN*-α, *STAT1*, *OAS1*, *Mx1*, and *PKR* in PK-15 cells with no CSFV infection. **(A–C)** PK-15 cells were treated with 0.1 μM MG132, same volume of DMSO, or left untreated. The mRNA levels of *IFN*-α **(A)**, *STAT1* **(B)**, *OAS1*, *Mx1*, and *PKR* **(C)** at 24 hpi were determined by qRT-PCR. Data in the bar plot are representative of three biological replicates. ns, not significant.

To explore the role of MG132 in the expression of the immune-related molecules in cells with no viruses, PK-15 cells were treated with MG132, the same volume of DMSO or left untreated. The relative mRNA levels of *IFN*-α, *STAT1*, *OAS1*, *Mx1*, and *PKR* were determined 24 h later. Results showed that MG132 had no obvious effect on expressions of *IFN*-α (Figure 7A), *STAT1* (Figure 7B), *OAS1*, *Mx1*, and *PKR* (Figure 7C).

## Discussion

Investigation of the pathogenic mechanism of CSFV has always been a hot topic. Interactions of viral proteins, such as N^pro^, E2, and NS3 of CSFV, with cellular proteins could result in blockage of innate immune pathways and downregulation of a variety of antiviral and inflammation-inducing proteins (Li S. et al., 2017; Lv et al., 2017; Goraya et al., 2018). The host has developed various ways to resist virus infection and replication, and the innate immune system is absolutely an important one. Autophagy is an intracellular degradation process and we have previously proved that CSFV utilized autophagy to ensure persistent replication (Pei et al., 2014). 26S proteasome, also in charge of protein degradation, has been identified to play significant roles in the replication of many viruses (Luo, 2016), while the impact of 26S proteasome on CSFV is poorly understood. In this study, we proved that MG132 could strongly inhibit the replication of CSFV (Figures 2, 4A), in which the upregulation of expressions of ISGs may play an important role (Figures 3B, 4C). MG132 induced the accumulation of STAT1 in the nucleus of uninfected cells nearby CSFV-infected cells (Figure 6), which may help keep the uninfected cell in a defensive state. Since 26S proteasome is in charge of the degradation of both cellular and viral proteins, MG132 may affect the immune system and viral replication at the same time. While MG132 does not directly affect the mRNA levels of ISGs in cells without CSFV (Figure 7), MG132 may regulate the expressions of ISGs through modulating the levels of related immune molecules. Viral proteins N^pro^, C, and p7 of CSFV have been reported to be degraded by 26S proteasome (Seago et al., 2010; Lin et al., 2014; Mine et al., 2015; Chen et al., 2019), and we have found that several other viral proteins are also affected by 26S proteasome (data not shown). Considering the relationships between CSFV viral proteins, the innate immune system and the 26S proteasome, the way that MG132 inhibits the replication of CSFV may need more detailed investigations.

In previous studies, different concentrations of MG132 are used to identify its role in viral replication. A study of the effect of MG132 on viability of MDBK cells showed that MG132 at a high concentration inhibits cell viability (Fiorito et al., 2017). In accordance, we identified that cell viability of PK-15 decreased with higher concentration of MG132 (Figure 1A). A study about hepatitis E virus (HEV) shows that the inhibitory role of high concentration of MG132 in viral replication was nonspecific considering the role of MG132 in widespread depression of expression of cellular proteins (Xu et al., 2015). It has been identified that MG132 at an even lower concentration of 5 nM could suppress HCV RNA replication by 85% without any influence on cell viability (Ujino et al., 2010). In this study, MG132 of high concentrations decreased CSFV replication and increased expressions of ISGs at the same time (Figure 4), indicating that the impact of MG132 on CSFV replication is specific.

MG132 has been identified to affect the replication of many viruses in various ways. MG132 suppresses HSV-1 replication by inhibiting activation of nuclear factor-κB (NF-κB), an important molecular of innate immune signaling pathway (La Frazia et al., 2006). Viral protein synthesis and virus replication of HCMV are both decreased when treated with MG132 (Kaspari et al., 2008). MG132 could attenuate the replication of HEV (Karpe and Meng, 2012). A study about PCV2 revealed that MG132 decreased viral protein expression and RNA transcription in a cell cycle-dependent manner (Cheng et al., 2014). In our study, we revealed a new mechanism that MG132 could affect the JAK-STAT pathway and the following transcription of several ISGs in CSFV-infected cells. The strong and specific inhibitory role of MG132 in CSFV replication may need further detailed investigation to see if the mechanism is virus-specific or universal.

NS4A is a small nonstructural protein of CSFV and studies about NS4A are limited up to now. In this study, we revealed that CSFV infection or viral NS4A protein could result in the downregulation of STAT1 protein (Figure 5). In CSFV-infected cells, NS4A protein might contribute to CSFV replication via downregulation of the level of STAT1 protein. Upregulation of pSTAT1 and downregulation of STAT1 in CSFV-infected cells may be a comprehensive result of the combat between the innate immune system and viral proteins including but not limited to NS4A. We found that CSFV NS4A protein could be degraded by the 26S proteasome (data not shown). Considering the extensive role of the 26S proteasome in the degradation of viral proteins of CSFV, the relation of 26S proteasome and CSFV should be close and complicated, which needs detailed investigation.

The combat between CSFV and the host is complicated. It has been identified that IFN-α and IFN-β could strongly inhibit CSFV replication, and CSFV has developed many ways to resist this. Viral proteins, N^pro^ and C, are reported to interfere with IFN-α or IFN-β secretion by interacting with cellular proteins within the host immune pathway (Ruggli et al., 2005; Doceul et al., 2008; Li et al., 2013). Upon sensing CSFV infection, cytokines are released followed by transcription of a series of ISGs induced by the activation of the JAK-STAT pathway. A previous study has confirmed the critical roles of type III interferons in the activation of the JAK-STAT pathway in CSFV-infected cells (Cai et al., 2017). In our study, significant innate immune molecules related to viral infection including IFN-α and three ISGs are examined. Consistent with previous studies, the inactivation of IFN-α may be a result of the inhibitory role of viral proteins. While the roles of viral proteins in the modulation of expressions of ISGs are not clearly identified, future studies may focus on the interplay between the JAK-STAT pathway and viral proteins considering the critical roles of ISGs in CSFV replication.

## Conclusion

In conclusion, we reveal that MG132 could strongly inhibit CSFV replication *in vitro*, in which the upregulation of ISGs induced by the JAK-STAT pathway might play an important role. Our study provides new insights into the mechanism associated with CSFV evasion of the innate immune system, as well as potential usage of MG132 in the cure of CSFV-infected pigs.

## Data Availability Statement

The raw data supporting the conclusions of this article will be made available by the authors, without undue reservation, to any qualified researcher.

## Author Contributions

YC, MZ, and JC conceived and designed the experiments. YC, SF, MZ, KW, EZ, SM, WH, SD, HX, JZ, HD, and LY performed the experiments and analyzed the data. YC, EZ, MZ, and JC wrote and revised the manuscript. All authors have read and approved the final manuscript.

## Conflict of Interest

The authors declare that the research was conducted in the absence of any commercial or financial relationships that could be construed as a potential conflict of interest.
